# Stochastic Dosimetry for the Assessment of Children Exposure to Uniform 50 Hz Magnetic Field with Uncertain Orientation

**DOI:** 10.1155/2017/4672124

**Published:** 2017-10-31

**Authors:** E. Chiaramello, S. Fiocchi, P. Ravazzani, M. Parazzini

**Affiliations:** Istituto di Elettronica e di Ingegneria dell'Informazione e delle Telecomunicazioni, CNR, Milano, Italy

## Abstract

This study focused on the evaluation of the exposure of children aging from five to fourteen years to 50 Hz homogenous magnetic field uncertain orientation using stochastic dosimetry. Surrogate models allowed assessing how the variation of the orientation of the magnetic field influenced the induced electric field in each tissue of the central nervous system (CNS) and in the peripheral nervous system (PNS) of children. Results showed that the electric field induced in CNS and PNS tissues of children were within the ICNIRP basic restrictions for general public and that no significant difference was found in the level of exposure of children of different ages when considering 10000 possible orientations of the magnetic field. A “mean stochastic model,” useful to estimate the level of exposure in each tissue of a representative child in the range of age from five to fourteen years, was developed. In conclusion, this study was useful to deepen knowledge about the ELF-MF exposure, including the evaluation of variable and uncertain conditions, thus representing a step towards a more realistic characterization of the exposure to EMF.

## 1. Introduction

Extremely low frequency magnetic fields (ELF-MF), such as magnetic fields generated by transmission of electricity power lines, are ubiquitous in daily life. Moreover, the continuous development of new technologies for energy transmission, such as wireless low frequency power transmission, the adaptions of the distribution network to more efficient standards, and the construction of new power lines, contribute to the raising of public awareness over the potential adverse health effects due to the interaction of ELF-MF with the human body.

The exposure to ELF-MF of high amplitude causes well known acute biological effects on the nervous system, such as nerve stimulation and induction of retinal phosphenes [1]. Furthermore, starting from the late 1970s, many studies focused on a possible association, firstly suggested by Wertheimer and Leeper [2], between long-term exposure to ELF-EMF and an increased risk of childhood cancer (see, e.g., [3]), with an overall conclusion that the exposure to low frequency magnetic fields could be associated with an increased risk of leukaemia in children. This last possibility led the International Agency for Research on Cancer (IARC) [4] to classify ELF magnetic fields as “possibly carcinogenic to humans” in 2002.

Many studies have investigated the assessment of the exposure to magnetic field in terms of induced electric fields within the body at the specific frequency of 50 Hz, also focusing on children [5, 6] and fetuses [5, 7–11], for their precocity of exposure. Although some of these studies focused on the assessment of how the exposure is influenced by the anatomy, age, and posture of the exposed subjects [5, 6, 11], they provided an assessment of the exposure limited to few specific exposure scenarios and, hence, they provide no information about how the exposure changes in realistic and highly variable scenarios. Such an assessment is indeed a challenging task, due to the intrinsic variability of the parameters that influence the exposure, as the morphology, the anatomy and the posture of the exposed subject, the dielectric properties that characterized the tissues of the subject, and the reciprocal position of the field and the exposed subject [12]. Two previous studies [13, 14] dealt with the task of providing a description of the exposure due to 50 Hz ELF-MF in realistic scenarios, specifically in fetuses. In particular, these studies assessed how the variability of both magnetic field orientation [13] and of dielectric properties assignment to the fetal tissues at different gestational ages [14] influence the estimation of the induced electric field due to ELF-MF exposure at 50 Hz in fetal tissues using stochastic dosimetry. Stochastic dosimetry is a method that combines electromagnetic computational techniques and statistics to build surrogate models that can be used to obtain, parsimoniously, the distribution of the quantity of interest (the induced electric field for instance), replacing by analytical equations the heavy numerical simulations that would be needed to characterize the highly variable exposure using only classical electromagnetic computational techniques. Among the statistical approaches that could be used to build surrogate models in stochastic dosimetry, the polynomial chaos (PC) theory [15] resulted in being an efficient tool to assess the variability of the EMF exposure both at radio frequency [16, 17] and at low frequency [13, 14]. Results obtained by [13, 14] showed that, while variations in the dielectric properties could not be established as a decisive factor for the exposure of fetuses, the variations in the orientation of the magnetic field strongly influenced the electric field induced in the fetal tissues.

Starting from these findings, the current study focused on the exposure to ELF-EMF in uncertain scenarios characterized by variability, thus scenarios in which a subject is exposed to sources that can be considered from her/his point of view as positioned in an unknown location and randomly distributed in the space. The investigation is performed by using stochastic dosimetry based on the PC theory, to evaluate how the variations in the orientation of the magnetic field at 50 Hz affect the electric fields induced in the tissues of children, estimating the fields in 6 high resolution models of children in the range of five to fourteen years. For each considered child, a PC surrogate model describing the electric field induced in each tissue of interest as a function of the orientation of the magnetic field was built. The electromagnetic metrics and the estimation procedure and details (e.g., identification of the target tissues) were set according to the ICNIRP International Committee of Non-Ionising Radiation Protection guidelines [18]. A “mean stochastic model,” obtained using as experimental data set the mean values of the exposure across all the children, was developed. This could be useful to estimate the level of exposure in each tissue of a representative child in the range of age from five to fourteen years.

## 2. Materials and Methods

**Figure 1:**
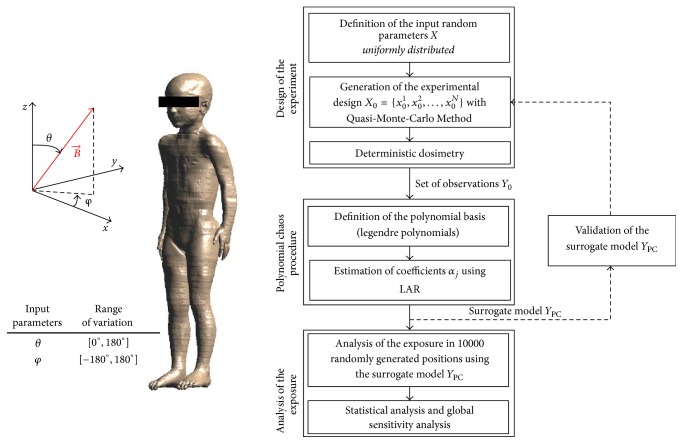
Schematic view of the exposure scenarios and flow chart of the experimental procedure.

Figure 1 shows a schematic view of the exposure scenarios (left side) and the flow chart of the experimental procedure (right side). The electric field induced in children tissues was assessed by varying the orientation of a perfectly homogeneous 50 Hz** B**-field of 200 *μ*T of amplitude, using surrogate models based on PC expansions. Each surrogate model describes how the variable of interest* Y* (i.e., the induced electric field) was affected by the variability in the input parameters* X* (i.e., the different orientation of the** B**-field). Three main steps composed the experimental procedure. The first step, namely, “design of the experiment,” consisted of using deterministic dosimetry, that is, dosimetry based on computational methods, for the evaluation of a set of *N* experimental observations *Y*_0_ of the variable of interest* Y*, needed for the construction of the surrogate models. The second step, namely, “polynomial chaos procedure,” focused on the development of a surrogate model *Y*_PC_ using the polynomial chaos method. The surrogate model thus obtained was validated with a cross-validation procedure, aimed at defining the minimum number *N* of experimental observations needed to achieve an acceptable solution, that is, to obtain a percentage mean square “leave-one-out” error lower than 5%. Finally, the obtained surrogate model was used in the third step, namely, “analysis of the exposure,” for the exposure assessment of each child. Details about each step are as follows.

### 2.1. Design of the Experiment

The random input vector *X* was defined as the two spherical angles theta (*θ*) and phi (*φ*), which characterized the** B**-field orientation (see Figure 1). To develop the PC models, it was necessary to quantify the source of uncertainty, thus hypothesizing the probability distribution of the input parameters *θ* and *φ*. In order to avoid losing generality, all the orientations of the *B*-field were considered as having the same probability and the only hypothesis made by the authors was about the ranges of variability of the input parameters. As stated by the maximum entropy principle, the least biased probability distribution representing the information of a random variable given only its boundaries is the uniform one [19]; thus the two input parameters were hypothesized to be uniformly distributed.

Analogously to the approach described by Liorni et al. [13], in order to take into account all possible differences in the amplitude of the electric fields induced in the tissues by** B**-fields of opposite directions that could arise from anatomical asymmetries, the exposure was assessed for all the possible orientation of the** B**-field in the 3-dimensional space. The ranges of variation of *θ*  [0,180°] and *φ*  [−180°, 180°] were set according to the convention of unique spherical coordinates.

The experimental design *X*_0_ was generated using a Quasi-Monte-Carlo method based on the Sobol function applied on the joint probability density function of the input parameters *X*_*i*_ [20].

In this study, the variable of interest *Y*, modelled by PC expansion, is the 99th percentile value of root mean square of each child tissue-specific induced electric field (indicated *E*^99th^) averaged on a 2 mm side cube. This metric is adopted by the International Commission of Non-Ionizing Radiation Protection guidelines [18] as the relevant tissue-specific value to be compared with the basic restrictions.

The quantity of interest *Y*_0_ (i.e., the *E*^99th^ values) was evaluated using deterministic dosimetry based on magnetoquasistatic low frequency solver implemented on the simulation platform SEMCAD X (Schmid & Partner Engineering), which is based on the Scalar Potential Finite Element (SPFE) method. In the low frequency range, where the maximum dimension of the computational domain is much smaller than the free space wavelength, the magnetic vector potential **A**, defined as

(1) $$\begin{array}{c} \nabla \times A=\frac{\partial B}{\partial t}, \end{array}$$

is decoupled from the electric field *E* and thus can be computed using Biot-Savart's law. Moreover, since in the human body the displacement current is neglected with respect to the conduction current for the exposure condition here studied (*σ* ≫ *jωε*), where *σ* and *ε* are the electrical conductivity and the permittivity of the tissues, respectively, and *ω* is the angular frequency of the field,* E* can be calculated from the scalar potential Φ by

(2) $$\begin{array}{c} -\nabla \cdot \sigma \nabla \Phi =j\omega \nabla \cdot \left(\;\sigma A\;\right), \end{array}$$

where the finite element method is used to solve for Φ. Rectilinear grids were applied to discretize the complex anatomical models with a grid resolution of 1 mm.

**Figure 2:**
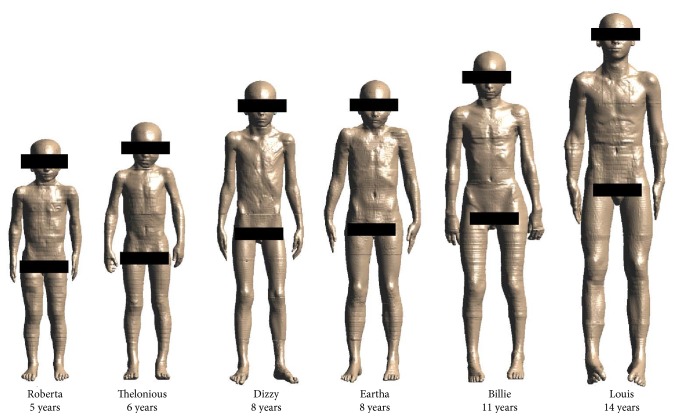
High resolution male and female children models with age ranging from five to fourteen years.

The simulations were conducted using six high resolution male and female children models with age ranging from five to fourteen years (see Figure 2) from the Virtual Family and the Virtual Classroom [21]. The dielectric properties (permittivity and conductivity values) in each tissue of the children were assigned according to the data available in literature [11, 22–24].

Following the ICNIRP guidelines [18], in this study for each model, *E*^99th^ induced in each tissue of the central nervous system (CNS) and in the peripheral nervous system (PNS), was evaluated. ICNIRP [18] defines CNS as “the portion of the vertebrate nervous system consisting of the brain and spinal cord, but not including the peripheral nerves” and PNS as “nerves found outside the central nervous system and leading to and from the central nervous system.” Taking into account these ICNIRP definitions, ten CNS tissues are included in the children models used in this study, that is, brain gray matter, brain matter, cerebellum, hippocampus, hypothalamus, medulla oblongata, midbrain, pons, spinal cord, and thalamus, whereas all the nerves of the whole body, including the optical nerves and the spinal nerves (i.e., those nerves connecting the spinal cord with the rest of the body, but not the spinal cord itself) are included in the PNS. The value of *E*^99th^ representative of the induced electric field in the whole CNS was defined as the highest among all the *E*^99th^ values evaluated in each of the CNS tissues. While the tissues of the CNS were similarly segmented for all the children models, some differences were found when considering the PNS. In particular, for different models, the peripheral nervous tissue included nerves in different parts of the body. More specifically, for Roberta, Thelonious, Dizzy, and Louis, it included spinal and optical nerves, for Billie it included spinal, optical, and lower limb nerves, and for Eartha it included only few lumbar nerves.

The children models were exposed to a perfectly homogeneous** B**-field at 50 Hz of 200 *μ*T of amplitude, with *N* different orientation described by the experimental set *X*_0_, to obtain the set of observation *Y*_0_ needed for the estimation of the coefficient of the PC expansion.

### 2.2. Polynomial Chaos Procedure

The polynomial chaos is a spectral method and consists in the approximation of the system output *Y* in a suitable finite-dimensional basis Ψ(*X*) made of orthogonal polynomials [20]. A truncation of this polynomial expansion can be as follows:

(3) $$\begin{array}{c} Y=M\left(\;X\;\right)=\overset{P-1}{\underset{0}{\sum }}\alpha_{j}\psi_{j}\left(\;X\;\right)+\varepsilon , \end{array}$$

where *Y* is the system output, *X* is the random input vector made of the input parameters *x*_*i*_, *ψ*_*j*_ are the multivariate polynomials belonging to Ψ(*X*), *α*_*j*_ are the coefficients to be estimated, *ε* is the error of truncation, and *P* is the size of the polynomial basis Ψ(*X*). Each multivariate polynomial *ψ*_*j*_ is built as tensor product of univariate polynomials orthogonal with respect to the probability density function of each input parameter *x*_*i*_.

The first step (Figure 1) in the PC procedure is the choice of the proper polynomial basis that would be used to build up the PC expansion. The proper univariate polynomials have to be orthogonal with respect to the probability density function of each input parameter *x*_*i*_ [20]. As the input parameters *x*_*i*_ were supposed to be uniformly distributed (see previous section), Legendre polynomials were used [25].

The second step (Figure 1) is the estimation of the coefficients *α*_*j*_ of the PC expansion: the chosen method was the Least Angle Regression (LAR) algorithm [26], which is based on least-square regression with respect to the series of *N* observations *Y*_0_ of the system output* Y* [27].

In this study, the surrogate models based on the PC theory were built using the Matlab based software “UQLab: The Framework for Uncertainty Quantification” [28]. For a more detailed description of the PCE theory and its applications in stochastic dosimetry, see [13, 17].

### 2.3. Validation of the Surrogate Model

**Figure 3:**
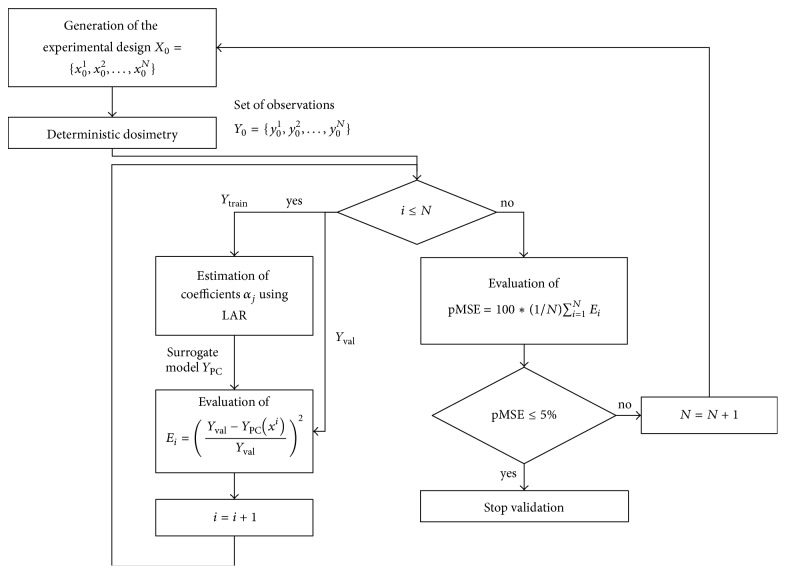
Schematic view of the leave-one-out cross-validation procedure.

The validation of the surrogate model (shown in Figure 3) was based on a leave-one-out cross-validation approach, a technique developed in statistical learning theory (see, e.g., [27]) and here used to reduce at minimum the size of the experimental design. To that purpose, the set of observation *Y*_0_, obtained with deterministic dosimetry from the experimental design of size *N*, was recursively divided into two subsets: *Y*_train_, containing all the observations except for the *i*th one, and *Y*_val_, containing only the excluded observation. A surrogate model *Y*_PC_ was built using the subset *Y*_train_ and then its prediction of the excluded *i*th point (*Y*_PC_(*x*^*i*^)) was compared with *Y*_val_, calculating the error *E*_*i*_ defined as

(4) $$\begin{array}{c} E_{i}={\left(\;\frac{Y_{val}-Y_{PC}\left(x^{i}\right)}{Y_{val}}\;\right)}^{2}. \end{array}$$

The percentage mean square “leave-one-out” error pMSE was defined as the sum of all the *E*_*i*_, normalized on the number of sample* N*:

(5) $$\begin{array}{c} pMSE=100*\frac{1}{N}\overset{N}{\underset{i=1}{\sum }}E_{i}. \end{array}$$

The procedure has been repeated increasing the size *N* of the experimental set and modifying the maximum degree *p* of polynomials *ψ*_*j*_ until the achievement of a pMSE lower than 5%. The starting dimension of the experimental design *N* was defined using the “thumb rule” described in [29]. The thumb rule defined by Berveiller [30] is an empirical rule to select the size *N* of the experimental design, namely, *N* = (*M* − 1)*P*, where *M* is the number of input variables (i.e.,* M* = 2) and* P* is maximum size of the polynomial basis, defined as *P* = (*M* + *p*)!/(*M*!*p*!), where *p* is the maximum degree of the polynomials. With* p* = 5, the value of *N* at the beginning of the cross-validation process was fixed equal to 21. At the end of the process, the pMSE threshold value of 5% was satisfied for a size of the experimental design *N* equal to 30 and a maximum degree *p* of the polynomials of the PC expansions equal to 5.

### 2.4. Analysis of the Exposure

Once all the PC models have been built for the induced *E*^99th^ in each specific tissue for all the children, several orientations of the** B**-field have been randomly selected using Quasi-Monte-Carlo method based on the Sobol function applied on the joint probability density function of the input parameters *x*_*i*_ [18]. As the computational effort in assessing the exposure using the PC models was very low, a very high number (i.e., 10000) of orientations of the** B**-field was considered, in order to cover the range of variations of the input parameters. The *E*^99th^ values relative to each specific orientation have then been calculated by means of the PC models. A statistical analysis has been performed to assess the variability of the exposure due to the change of the orientation of the** B**-field, in terms of Quartile Coefficient of Dispersion (QCD), calculated as

(6) $$\begin{array}{c} QCD=\frac{Q_{3}-Q_{1}}{Q_{3}+Q_{1}}, \end{array}$$

where *Q*_1_ and *Q*_3_ are, respectively, the first and the third percentiles of the distribution of *E*^99th^ obtained for the several orientation.

Moreover, for each of the considered tissues of CNS, for the whole CNS and for PNS, an analysis of the** B**-field orientation that induced *E*^99th^ values higher to 90% of their maximum value was performed.

A global sensitivity analysis to assess how the variability of each single input parameter (i.e., the two spherical coordinates of the** B**-field) influenced the output (i.e., *E*^99th^) was carried out. The global sensitivity analysis was performed by means of a variance-based method introduced by Sobol [31], consisting in decomposing the variance of the system output as a sum of contributions of each input parameter and thus calculating the Sobol indices as the ratios between the partial variances of the input parameters and the total variance of the system output. The Sobol indices, computed for each input parameter directly from the surrogate models (for more details, see [29]), were normalized with respect to the sum of all the Sobol indices under consideration.

### 2.5. Mean Stochastic Model

In order to obtain a description of which is the level of exposure of a “mean child” in the range of age from five to fourteen years old, a “mean stochastic model” was developed, with the procedure described in the following. First, the experimental values *E*^99th^ of the observations sets *Y*_0_ obtained with deterministic dosimetry using the six children models were averaged, obtaining a “mean” set of observation *Y*_0_m*e*an_. Then, starting from *Y*_0_m*e*an_, the polynomial chaos procedure was applied to obtain the mean surrogate model *Y*_PC_m*e*an_. Finally, *Y*_PC_m*e*an_ was used to estimate the *E*^99th^ values relative to 10000 possible orientations of the** B**-field.

## 3. Results

**Figure 4:**
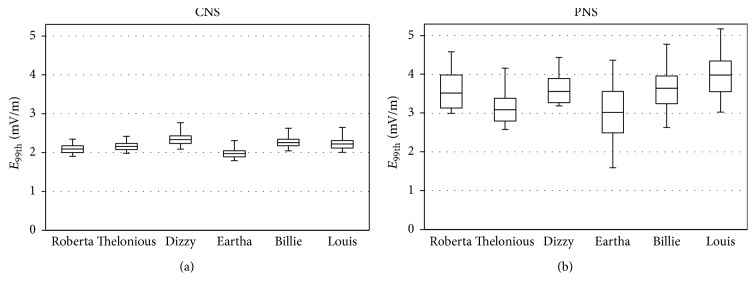
Box plots of *E*^99th^ in the (a) CNS and (b) PNS tissues for all the children models.

Figure 4 shows the box plot of distribution of the 10000 values of *E*^99th^ induced in the CNS (Figure 4(a)) and PNS (Figure 4(b)) tissues of the six children models (the lower and upper bound of the box represent the first and the third quartiles, the line is the median value and the whiskers are the minimum and maximum value). For both CNS and PNS tissues, values of *E*^99th^ were found to be almost equal across the six children, with median values in the range 2.0–2.3 mV/m for CNS and in the higher range 3-4 mV/m for PNS and maximum values in the range 2.3–2.8 mV/m for CNS and in the range 4.2–5.2 mV/m for PNS. The variability of the exposure in terms of *E*^99th^ in the CNS due to the change in the orientation of the** B**-field was low for all the children models, resulting in QCD values lower than 5%. For the PNS, the variability of the exposure was slightly higher, resulting in QCD values in the range 10–18%, with the lowest value for Dizzy and the highest value for Eartha. The results of the global sensitivity analysis, reported in Table 1, showed that the variability of the two input parameters influenced the induced electric field in different ways for the CNS and PNS tissues. More specifically, for CNS tissues, the normalized Sobol indices showed that for all the children models the parameter *θ* influenced *E*^99th^ for almost 80% of the total variation, respectively, while *φ* influenced *E*^99th^ for only the remaining 20%. For PNS tissues, the normalized Sobol indices showed that, for the youngest children, that is, Roberta, Thelonious, and Dizzy, the parameter *θ* influenced *E*^99th^ for most of the total variation, with normalized Sobol indices equal to 98%, 83%, and 95%, while the parameter *φ* accounted for the remaining 2%, 17%, and 5% of the variability. Also for Eartha and Louis the normalized Sobol indices showed that the parameter *θ* was the most influential on the variability of *E*^99th^, accounting for about 63% of the total variability, while for Billie the two parameters *θ* and *φ* were very equally influential on *E*^99th^, accounting for 47% and 53% of the total variation, respectively.

**Figure 5:**
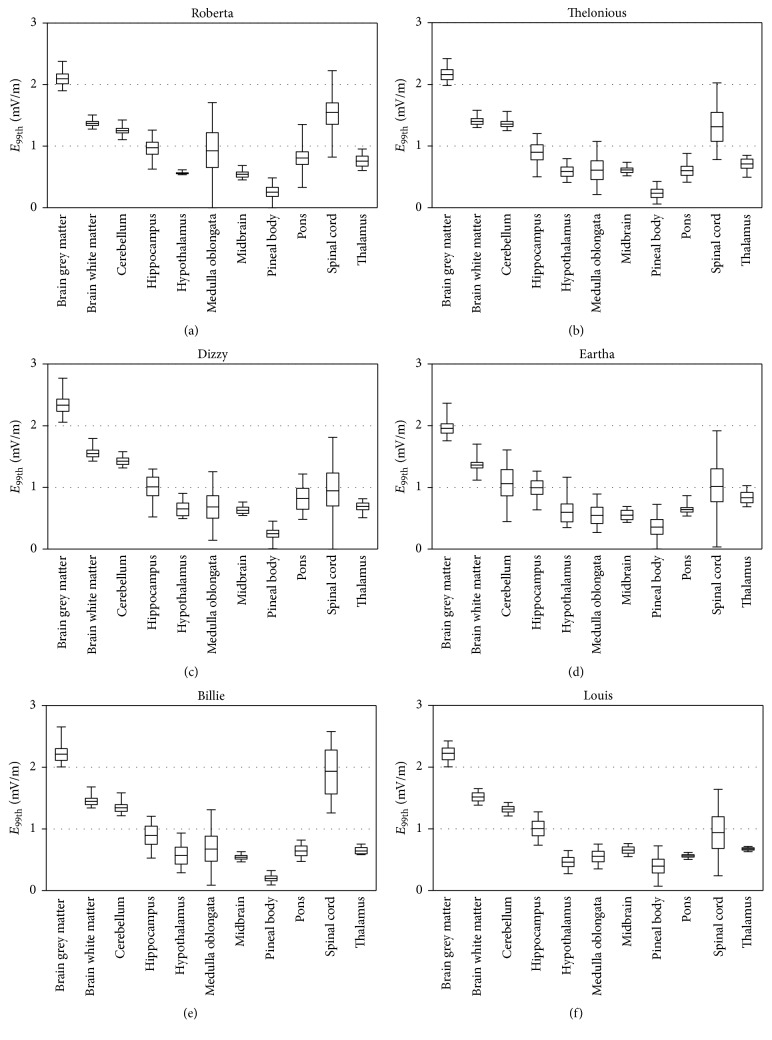
Box plots of *E*^99th^ in each tissue belonging to CNS for different children models (a) Roberta, (b) Thelonious, (c) Dizzy, (d) Eartha, (e) Billie, and (f) Louis. The lower and upper bound of the box represent the first and the third quartiles, the line is the median value, and the whiskers are the minimum and maximum values.

Figure 5 shows the descriptive statistic as box-plot of the of the *E*^99th^ values induced in each of the ten tissues that belong to the CNS obtained in 10000 random orientation of the** B**-field, for all the considered children models. As a general observation, the values of *E*^99th^ found in the same tissue across the children models were similar. In particular, in each child the highest median and maximum values of *E*^99th^ were observed in the brain grey matter, brain white matter, and spinal cord tissues, while the lowest median and maximum values of *E*^99th^ were observed in the midbrain. The variability of *E*^99th^ due to the change in the orientation of the** B**-field, expressed as QCD, was similar across the children models and varied from tissue to tissue. In particular, in all the children we found minimum QCD values equal to about 3% for brain grey matter and brain white matter and maximum QCD values equal to about 25% for spinal cord and cerebellum tissues.

**Figure 6:**
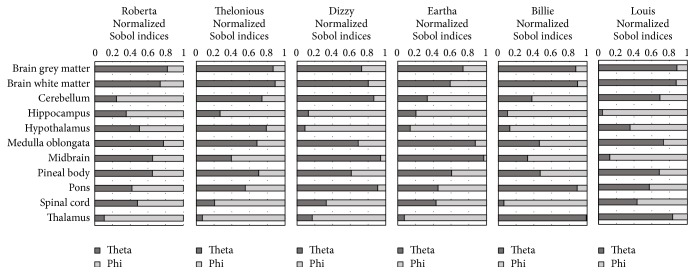
Normalized Sobol indices found for *E*^99th^ in each of the ten tissues belonging to CNS.

The global sensitivity analysis was performed for *E*^99th^ in each of the ten tissues, resulting in the normalized Sobol indices reported in Figure 6. Results showed that the two input parameters, that is, *θ* and *φ*, influenced the exposure in terms of *E*^99th^ differently in each tissue, but similarly across the different children models. In particular, for most children (except for Billie and Louis), the normalized Sobol indices showed that the parameter *θ* influenced *E*^99th^ for the most of the total variation in brain grey matter, brain white matter, medulla oblongata, and midbrain (in the range 65–95%), while the parameter *φ* influenced *E*^99th^ for the most of the total variation in hippocampus, hypothalamus, and thalamus (in the range 64–93%). For the remaining tissues, both parameters were equally influential on the induced electrical field in terms of *E*^99th^.

**Figure 7:**
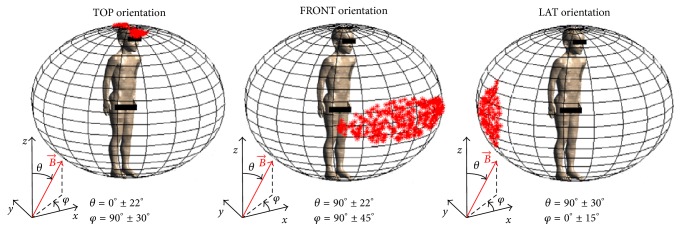
Examples, plotted as distribution on a unitary sphere, of those orientations of** B**-field that induced *E*^99th^ values higher than the 90% of the maximum value of *E*^99th^, in the neighborhood of the orthogonal orientation parallel to the vertical axis (“TOP” orientation), the anteroposterior axis (“FRONT” orientation), and the mediolateral axis (“LAT” orientation) of the body. For each pattern, values of *θ* and *φ* corresponding to the** B**-field orientation that induced electric field higher than the 90% of the maximum value of *E*^99th^ are reported.

For each of the considered tissues of CNS, for the whole CNS and for PNS in each children model, an evaluation of which, among the 10000 considered orientations of the** B**-field, induced *E*^99th^ values higher to 90% of their maximum value was assessed. Results showed that this condition was satisfied in the neighborhood of the orthogonal orientations of** B**-field along the vertical, anteroposterior and mediolateral axes of the body.  Figure 7 shows examples, represented as distribution on a unitary sphere, in which the orientations of** B**-field that induced electric field higher than the 90% of the maximum value of *E*^99th^ were in the neighborhood of the** B**-field orientation parallel to the vertical axis (“TOP” orientation), the anteroposterior axis (“FRONT” orientation), and the mediolateral axis (“LAT” orientation) of the body. For each panel of the figure, the values of *θ* and *φ* describing the range of** B**-field orientation satisfying the above conditions are reported. It should be noted that, even if not shown in Figure 7 for the sake of clarity, orientations of** B**-field that induced electric field higher than the 90% of the maximum value of *E*^99th^ were found also on the opposite side of the presented sphere, in symmetrical positions. Thus, the notations “TOP,” “FRONT,” and “LAT” orientations will be used in the following to indicate the* B*-field orientations parallel to the three axes of the body considering both signs of the vector (i.e., “TOP” orientation corresponded to both top-to-bottom and bottom-to-top polarizations, “FRONT” orientation corresponded to both front-to-back and back-to-front polarizations, and “LAT” orientation corresponded to both left-to-right and right-to-left polarizations).

Table 2 shows, for each tissue and each child, the orientations of the** B**-field that induced *E*^99th^ values higher to 90% of the maximum value in that tissue represented as the ranges of variation of *θ* and *φ* with respect to the three orthogonal orientations of** B**-field (i.e., TOP, FRONT, and LAT orientation). These ranges of variations represent the “width” of the neighborhood around each orthogonal orientation. As a general observation, the orientations of** B**-field for which the induced electric field was found to be higher were different from tissue to tissue. For the whole CNS and for the brain grey matter tissues,** B**-field orientation that induced highest values of *E*^99th^ were found around TOP, FRONT, and LAT orientation for all the children, while for most of the remaining tissues only one or two orientation patterns were present, with some difference between children. The higher *E*^99th^ value was found around “TOP” orientation for PNS (for all the children except than for Billie), brain matter, cerebellum, midbrain, pons, and thalamus. The “FRONT” orientation pattern was found in brain white matter (for Roberta, Eartha, and Louis), hippocampus, and thalamus (for all the children except for Billie). Finally, the “LAT” orientation pattern was found in cerebellum, hypothalamus, medulla oblongata, pons, and spinal cord. It should be noticed that, analogously to previous observations about Figure 7, the same ranges of variations were found for both each considered orthogonal orientation and its symmetrical orientation. Thus, as an example, the ranges reported for the “TOP” orientation were referred to the neighborhood of both the top-to-bottom and bottom-to-top polarizations.

### 3.1. Mean Stochastic Model

**Figure 8:**
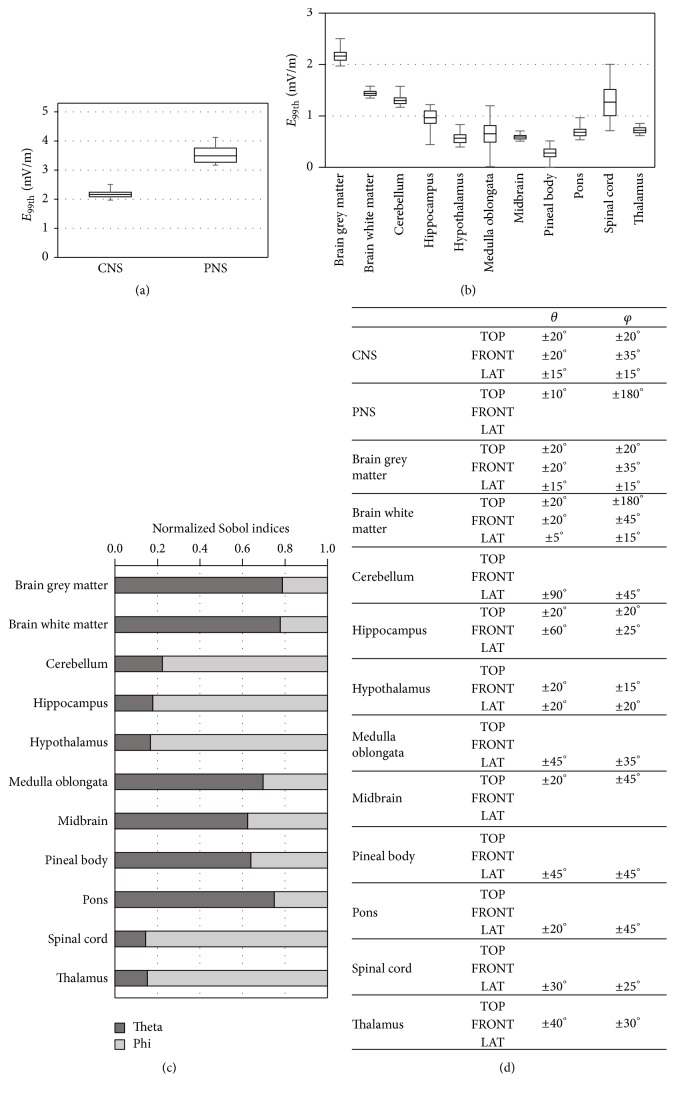
Results obtained with the mean stochastic model. (a) Statistic descriptive of *E*^99th^ in CNS and PNS. (b) Statistic descriptive of *E*^99th^ in each tissue belonging to CNS. (c) Normalized Sobol indices found for *E*^99th^ in each of the 10 tissues belonging to CNS. (d) Orientations of the** B**-field that induced in each tissue *E*^99th^ values higher to 90% of the maximum value in that tissue.

In order to obtain a description of which is the level of exposure of a “mean child” in the range of age from five to fourteen years old, a “mean stochastic model” was developed and used to evaluate the exposure in terms of *E*^99th^ for 10000 possible orientations of the** B**-field.  Figure 8 shows results obtained with the mean stochastic model, as box plot of distribution of the 10000 values of *E*^99th^ induced in the CNS and PNS (Figure 8(a)) and in each specific tissue belonging to the CNS (Figure 8(b)). The distribution of *E*^99th^ showed median and maximum values equal to 2.2 mV/m and 2.5 mV/m, for CNS, and equal to 3.5 mV/m and 4.1 mV/m, for PNS. The variability of the exposure in terms of *E*^99th^ due to the change in the orientation of the** B**-field resulted in QCD values equal to 4% and 7%, for CNS and PNS, respectively. Results of the global sensitivity analysis, reported in Table 1, showed that the parameter *θ* influenced *E*^99th^ in both CNS and PNS for most of the total variation, while *φ* was less influential. Considering each tissue of the CNS separately (Figure 8(b)) we found *E*^99th^ median values in the range 0.2–2.2 mV/m, with the highest values in brain grey matter, brain white matter, and spinal cord tissues and the lowest value in hypothalamus tissue. The variability of *E*^99th^ with the change of the orientation of the** B**-field, expressed as QCD, varied from tissue to tissue. In particular, minimum QCD values were equal to about 3% for brain grey matter and brain white matter and maximum QCD values were equal to about 20% for spinal cord. The global sensitivity analysis was performed for *E*^99th^ in each of the 10 tissues, resulting in the normalized Sobol indices reported in Figure 8(c). Results showed that the two input parameters, that is, *θ* and *φ*, influenced the exposure in terms of *E*^99th^ differently in each tissue. In particular, normalized Sobol indices showed that the parameter *θ* influenced *E*^99th^ for the most of the total variation in brain grey matter, brain white matter, medulla oblongata, midbrain, and pons, while the parameter *φ* influenced *E*^99th^ for most of the total variation in all the remaining tissues. Analogously to results previously shown for each child model, Figure 8(b) shows, for each tissue of the “mean child,” the range of orientations of the** B-**field that induced *E*^99th^ values higher to 90% of the maximum value in that tissue around the TOP, FRONT, and LAT orientation. For the whole CNS and for the brain grey matter and the brain white matter all the three orientation patterns were present. The higher *E*^99th^ values for PNS, hippocampus and midbrain were found around the “TOP” orientation, for hippocampus, hypothalamus, and thalamus around the “FRONT” orientation, and, finally, for cerebellum, hypothalamus, medulla oblongata, pons, and spinal cord around the “LAT” orientation.

## 4. Discussion

This study focused on the assessment of children exposure to a homogeneous magnetic field at 50 Hz of 200 *μ*T of amplitude with uncertain orientation. Most of previous studies assessing the exposure to homogeneous magnetic fields due to common ELF-MF sources as power lines, modelled them as uniform magnetic fields polarized in three orthogonal directions (see, e.g., [7, 8, 10, 13, 22]), discarding all the other possible orientations. In this study, the combined use of deterministic dosimetry and polynomial chaos theory allowed obtaining a complete description of the level of exposure in 10000 possible orientations of the** B**-field. We investigated the exposure in six high resolution anatomical models of children aging from five to fourteen years, evaluating the electric field induced in each tissue of the central nervous system (CNS) and in the peripheral nervous system (PNS), coherently with the ICNIRP guidelines [18].

As a first finding, for both CNS and PNS tissues, we found maximum values of *E*^99th^ almost equal across the six children, in the range 2.3–2.8 mV/m for CNS, and in the range 4.2–5.2 mV/m for PNS, when considering a magnetic field of 200 *μ*T. These values were significantly below the International Commission of Non-Ionizing Radiation Protection (ICNIRP) basic restrictions for the general public exposure [18], equal to 0.02 V/m for CNS tissues and equal to 0.4 V/m for PNS tissues. These results were coherent with previous findings by Bakker et al. [6], who found that the electric fields induced in the same children models used in this study when exposed to uniform magnetic fields at the ICNIRP reference levels were within the ICNIRP basic restrictions. When considering the induced electric field in each CNS tissues, we found similar results for all the considered child models. As expected, the highest median values of *E*^99th^ were found in the biggest tissues among those considered, that is, brain grey matter, brain white matter, and spinal cord tissues, while the lowest median maximum values of *E*^99th^ were found in the hypothalamus, that is, one of the smallest tissues considered. For all the child models, the highest values of *E*^99th^ were observed in the brain grey matter tissue, coherently with previous findings by Bakker et al. [6].

The variation of the orientation of the magnetic field influenced the exposure differently from tissue to tissue: for brain grey matter and brain white matter QCD values were low, equal to about 3%, while for other tissues, such as spinal cord and PNS, QCD values were equal to up to 25%. This is coherent with previous findings by Bakker et al. [6], who investigated the level of exposure only for Roberta model when considering few nonorthogonal orientations of a 50 Hz magnetic field, finding a variation in the *E*^99th^ values equal to about 20% in specific organs and tissues. The global sensitivity analysis showed that the influence of the two parameters describing the orientation of the magnetic field varied from tissue to tissue, thus highlighting that it was crucial to consider both parameters to obtain surrogate models able to reliably describe the level of exposure in the different tissues.

The analysis of which, among the 10000 considered orientations of the** B**-field, induced *E*^99th^ values higher to 90% of the maximum value showed that the highest values of *E*^99th^ were obtained in a neighborhood of the orthogonal orientations of** B**-field along the vertical, anteroposterior, and mediolateral axes of the body. This is coherent with previous findings by Liorni et al. [13], who found that the highest induced electric field in the fetus whole-body at three, seven, and nine months of gestational age was found when the orientation of** B** was in a region around the orthogonal orientations along the main axes of the mother body. Different patterns were found, varying from tissue to tissue: for the brain grey matter, which had an almost spherical shape for all the children, any prevalent** B**-field orientation was found, as values of *E*^99th^ higher than 90% of the maximum value were found for orientations of** B** in the neighborhood of all the three orthogonal orientations. On the contrary, for spinal cord, which showed a more elongated shape, the orientation of** B**-field which induced the highest values of *E*^99th^ was along the mediolateral axis of the body. This highlighted that the orientations of the** B**-field that induced the highest *E*^99th^ values strongly depended on the shape, the position, and the size of the tissue.

The small differences in the induced electric fields for children of different ages showed that, even if the range of age was wide, between five and fourteen years, the level of exposure in each tissue of the CNS was almost the same across the children. This may be due to the fact that, even if there are evident differences between the models as to the height and the weight [21], the sizes of CNS tissues were not so different (e.g., the variation of the volume of the brain grey matter between the five and the fourteen years old children, i.e., the youngest and the oldest considered children, was equal to only the 5%). Analogously, when considering the PNS, results were found to be quite similar for all the children, except for Billie. In particular, the main differences between Billie and the other children were found in the results of the global sensitivity analysis (for Billie the two parameters *θ* and *φ* were very equally influential on *E*^99th^, while for the other children the parameter *θ* was much more influential than *φ*) and in the identification of those orientations that induced the highest values of *E*^99th^ (for Billie the pattern representative of the orientations of** B**-field that induced the highest *E*^99th^ values was the “LAT” one, while for the other children it was the “TOP” one). These results were probably due to the fact that the peripheral nervous tissue in Billie included spinal, optical, and lower limb nerves, while in the other models it included only the spinal and optical nerves; thus the observed differences might not be representative of real differences due to the age of the children.

Therefore, it was possible to conclude that no significant difference was highlighted in the level of exposure of children of different ages when considering 10000 possible orientations of the magnetic field. This result is in line with the findings of previous studies [5, 6], in which the authors did not find a consistent pattern as a function of age in the exposure to ELF-MF of children of different age.

Starting from these findings, a “mean stochastic model” was developed, that is, a surrogate model obtained applying the polynomial chaos procedure to the mean values of the exposure found for the children models. This “mean stochastic model” was used to estimate the level of exposure in each tissue of a “mean child” in the range of age from five to fourteen years old. Results showed that, considering 10000 different orientations of the 50 Hz** B**-field, the electric field induced in CNS and PNS tissues of a generic child in the range of age from five to fourteen years was within the ICNIRP basic restriction for general public and that the variation of the orientation of the magnetic field influenced the exposure differently from tissue to tissue.

## 5. Summary

The main outcome of this study was the assessment of the children exposure to a 50 Hz homogenous magnetic field with variable and uncertain orientation. Considering such a source allowed modeling the exposure due to very common sources, such as electricity transmission and distribution networks in far field conditions in a more realistic way compared to previous studies. The use of the innovative approach of stochastic dosimetry allowed describing the exposure for a huge number of possible orientations of the B-field with a low computational effort. Results showed that the induced electric fields were within the ICNIRP basic restrictions for general public exposure in all cases. The variation of the orientation of the magnetic field influenced the exposure, causing QCD values up to 25% in specific tissues, highlighting that a proper assessment should not be limited to the orthogonal orientation of the** B**-field. No significant difference was found in the level of exposure of children of different ages when considering 10000 possible orientations of the magnetic field. A “mean stochastic model” for the assessment of the exposure in each tissue of a “mean child” in the range of age from five to fourteen years, useful for future investigations, was developed. In conclusion, results of this study added further knowledge about ELF-MF exposure, including the evaluation of variable and uncertain conditions, thus representing a step towards a more realistic characterization of the exposure to EMF.

## Figures and Tables

**Table 1:** Normalized Sobol indices for *E*^99th^ for CNS and PNS tissues.

	Normalized Sobol indices			
	CNS		PNS	
	*θ*	*φ*	*θ*	*φ*
Roberta	0.82	0.18	0.98	0.02
Thelonious	0.87	0.13	0.83	0.17
Dizzy	0.78	0.22	0.95	0.05
Eartha	0.83	0.17	0.63	0.37
Billie	0.87	0.13	0.47	0.53
Louis	0.88	0.12	0.64	0.36
Mean stochastic model	0.80	0.20	0.97	0.03

**Table 2:** Orientations of the magnetic field **B** thatinduced in each tissue *E*^99th^ values higher to 90% of the maximum value in that tissue. For each case, if one among the patterns “TOP,” “FRONT,” and “LAT” was present, the corresponding cell is filled with the ranges of variation of *θ* and *φ* with respect to the corresponding orthogonal orientations of **B**-field.

		Roberta		Thelonious		Dizzy		Eartha		Billie		Louis	
		*θ*	*φ*	*θ*	*φ*	*θ*	*φ*	*θ*	*φ*	*θ*	*φ*	*θ*	*φ*
CNS	TOP	±22°	±30°	±22°	±30°	±20°	±30°	±20°	±30°	±20°	±30°	±10°	±30°
	FRONT	±22°	±45°	±25°	±45°	±5°	±25°	±20°	±35°	±20°	±35°	±20°	±35°
	LAT	±22°	±15°	±22°	±10°	±5°	±10°	±25°	±40°	±25°	±15°	±25°	±10°

PNS	TOP	±15°	±180°	±20°	±180°	±10°	±180°	±10°	±40°			±20°	±180°
	FRONT												
	LAT									±25°	±30°		

Brain grey matter	TOP	±22°	±30°	±22°	±30°	±20°	±30°	±20°	±30°	±20°	±30°	±10°	±30°
	FRONT	±22°	±45°	±25°	±45°	±5°	±25°	±20°	±35°	±20°	±35°	±20°	±35°
	LAT	±22°	±15°	±22°	±10°	±5°	±10°	±25°	±40°	±25°	±15°	±25°	±10°

Brain white matter	TOP	±20°	±180°	±20°	±180°	±20°	±180°	±22°	±180°	±22°	±180°		
	FRONT	±25°	±45°					±25°	±35°			±25°	±35°
	LAT											±25°	±35°

Cerebellum	TOP			±20°	±30°	±20°	±20°	±40°	±30°				
	FRONT												
	LAT	±90°	±50°	±20°	±50°	±20°	±60°			±90°	±50°	±20°	±50°

Hippocampus	TOP												
	FRONT	±90°	±25°	±90°	±25°	±90°	±25°	±90°	±25°	±90°	±25°	±90°	±25°
	LAT												

Hypothalamus	TOP												
	FRONT	±20°	±40°										
	LAT	±15°	±15°	±30°	±50°	±50°	±30°	±50°	±30°	±90°	±30°	±30°	±50°

Medulla oblongata	TOP												
	FRONT												
	LAT	±45°	±25°	±30°	±15°	±45°	±20°	±40°	±20°	±90°	±25°	±25°	±40°

Midbrain	TOP	±20°	±45°	±30°	±45°	±15°	±180°	±20°	±180°	±35°	±35°	±30°	±30°
	FRONT												
	LAT												

Pons	TOP									±20°	±50°	±30°	±50°
	FRONT												
	LAT	±30°	±25°	±30°	±40°	±30°	±50°	±10°	±50°				

Spinal cord	TOP												
	FRONT												
	LAT	±30°	±25°	±35°	±25°	±35°	±30°	±40°	±30°	±40°	±30°	±30°	±30°

Thalamus	TOP	±15°	±20°	±25°	±20°	±25°	±20°	±15°	±20°				
	FRONT	±30°	±20°	±25°	±10°	±30°	±30°	±35°	±20°			±20°	±40°
	LAT									±30°	±90°		
